# The evaluation of scholarship in academic promotion and tenure processes: Past, present, and future

**DOI:** 10.12688/f1000research.16493.1

**Published:** 2018-10-05

**Authors:** Lesley A. Schimanski, Juan Pablo Alperin

**Affiliations:** 1 ScholCommLab, Simon Fraser University, Vancouver, BC, V6B 5K3, Canada; 2 School of Publishing, Simon Fraser University, Vancouver, BC, V6B 5K3, Canada

**Keywords:** promotion, tenure, incentives, academia, higher education, publishing

## Abstract

Review, promotion, and tenure (RPT) processes significantly affect how faculty direct their own career and scholarly progression. Although RPT practices vary between and within institutions, and affect various disciplines, ranks, institution types, genders, and ethnicity in different ways, some consistent themes emerge when investigating what faculty would like to change about RPT. For instance, over the last few decades, RPT processes have generally increased the value placed on research, at the expense of teaching and service, which often results in an incongruity between how faculty actually spend their time vs. what is considered in their evaluation. Another issue relates to publication practices: most agree RPT requirements should encourage peer-reviewed works of high quality, but in practice, the value of publications is often assessed using shortcuts such as the prestige of the publication venue, rather than on the quality and rigor of peer review of each individual item. Open access and online publishing have made these issues even murkier due to misconceptions about peer review practices and concerns about predatory online publishers, which leaves traditional publishing formats the most desired despite their restricted circulation. And, efforts to replace journal-level measures such as the impact factor with more precise article-level metrics (e.g., citation counts and altmetrics) have been slow to integrate with the RPT process. Questions remain as to whether, or how, RPT practices should be changed to better reflect faculty work patterns and reduce pressure to publish in only the most prestigious traditional formats. To determine the most useful way to change RPT, we need to assess further the needs and perceptions of faculty and administrators, and gain a better understanding of the level of influence of written RPT guidelines and policy in an often vague process that is meant to allow for flexibility in assessing individuals.

## Introduction

There is some question as to whether the academic system, and its means of evaluating the worth of its faculty’s contributions, has kept pace with the rapid evolution of technology and communications (e.g., Genshaft *et al*., 2016; Howard, 2013; Piwowar, 2013; Sanberg *et al*., 2014), as well as with societal goals such as ensuring equal opportunities for employment and career advancement regardless of gender, ethnicity, or other personal characteristics (e.g., Johnsrud & Jarlais, 1994; López *et al*., 2018; Menges & Exum, 1983; Whittaker *et al*., 2015). Some common complaints about academia, including those focused on lack of reproducibility ( Open Science Collaboration, 2015), problems with peer review ( Ross-Hellauer, 2017; Smith, 2006; Tennant *et al*., 2017), and the lack of access to research, could conceivably be reduced by building mechanisms that capitalize on freely available online communications and information-sharing tools. There is a clear desire by some, if not many, to make changes to the ways in which we organize academic activity and the dissemination of its products (e.g., Ellison & Eatman, 2008; O’Meara, 2014; O’Meara *et al*., 2015; Sid & Richardson Foundation Forum, 1997). However, there are barriers to change.

Chief amongst these barriers are the incentive structures currently in place for faculty career advancement. As Buttliere (2014) pointed out, “the problem is an ineffective reward system which makes doing the prosocial action … bad for the individual because it less efficiently achieves high impact work and thus promotion” (p. 1). To address the problems of academia, and conceptualize how its scholarly communication system might be improved to promote the active sharing of information and support a more efficient and transparent approach to conducting research, it is essential to understand the explicit rules of the game. To aid in this understanding, this paper offers a synthesis of the literature on review, promotion, and tenure (RPT) practices in the United States and Canada.

## Identifying RPT issues and areas for reform

The literature shows that there has been discontent, especially on the part of faculty but also in administrators, regarding the methods used to evaluate faculty for tenure and promotion (e.g., Diamond & Adam, 1998; Gordon, 2008; Harley *et al*., 2010). The major concerns about the RPT process can be grouped into two themes. Firstly, many faculty experience a dissonance between the apparent focus of RPT evaluation on research and publication, versus their actual work responsibilities, which often result in spending over half of their work hours on teaching (e.g., Diamond & Adam, 1998). Some also spend a great deal of time on service activities, which are barely recognized in the RPT process (e.g., Foos *et al*., 2004; Mamiseishvili *et al*., 2016). Secondly, faculty are concerned about the amount or type of publishing that is expected of them, the way their published works are assessed, and that the venues in which they are expected to publish (i.e., prestigious international and national journals, and university presses) don't have the capacity to support the amount of publication that universities want from their faculty (e.g., Adler *et al*., 2009; Brembs *et al*., 2013). Both of these factors can lead to frustration for university faculty.

That both of these concerns involve the publishing of research is not a coincidence: the challenges in scholarly communications and those of career advancement are intricately linked. To reduce the incongruence experienced by those wanting to both appropriately communicate their scholarship *and* advance successfully in their careers, it is necessary to understand the process that rewards activities within academia, especially as it pertains to publishing practices. A greater understanding of the RPT process may reveal an effective and efficient means for change. Changing the RPT process might lead to a reduction of the reliance on publication prestige or easily manipulated citation metrics, a restructuring of the peer review system, and even to an improvement in the quality, affordability, and flexibility of format in publishing venues. We begin by examining research that discusses how research, teaching, and service are viewed in RPT, and how the process places an emphasis on research and publications.

## Research, teaching, and service in the review process

In general, candidates for tenure and promotion are judged based on their research and publications, teaching effectiveness, and service. Although explicit weights for each aspect are not typically provided in RPT guidelines and policies, most faculty, across disciplines, assume that a strong research and publication record is necessary, and lack thereof cannot be compensated for by excellence in teaching and service ( Green, 2008; Harley *et al*., 2010; Youn & Price, 2009). This pattern has remained consistent since at least the 1990s.

Tenure and promotion requirements have changed over time: in the 1980s most university departments wanted to see excellence in at least one of research, teaching, or service ( Gardner & Veliz, 2014), and then a shift occurred in which excellence in teaching and service was no longer sufficient to earn tenure ( Youn & Price, 2009). By the 2000s, excellence in all three was expected with the most focus placed on research. This trend began to elicit concerns: in a 1989 survey of 5000 faculty (from two- and four-year U.S. academic institutions, across a spectrum of different disciplines), 68% agreed with the statement, “At my institution we need better ways, besides publications, to evaluate the scholarly performance of the faculty” ( The Carnegie Foundation for the Advancement of Teaching, 1989; p. 52). Although 71% of these faculty preferred teaching to research, publishing was considered a dominant factor in determining faculty career success ( The Carnegie Foundation for the Advancement of Teaching, 1989). Similarly, in 1989-1990, the Higher Education Research Institute surveyed over 35 000 faculty who taught undergraduate courses, representing all categories of higher education institutions in the U.S., and 44% of faculty at public universities felt that institutional demands for research productivity interfered with their ability to teach effectively ( Astin *et al*., 1991).

Fairweather (1993) analyzed surveys of over 4000 faculty in four-year colleges and universities and found that research productivity best predicted success in promotion, tenure, and salary increases across institution types and disciplines. Teaching was rarely a contributing factor to RPT success, and, in some cases, salary appeared to be negatively influenced by teaching hours. Commentary at the time reflected these ideas: an article appeared in The Chronicle of Education entitled “Teaching Awards: Aid to Tenure or Kiss of Death?” and another article commented, “Some professors … regard the Teacher of the Year Award as the kiss of death … I personally know three different professors at three different institutions who have gotten the Teacher of the Year Award and were then told that their contracts would not be renewed” ( Sowell, 1990, p. 69).

### Perceptions of the shift towards prioritizing research in career advancement

Some have theorized that faculty focus on research is necessary for the advancement of knowledge generation and thus should be the most important and valued aspect of an academic career ( Zuckerman & Merton, 1972). Correspondingly, an investigation in 49 research and doctoral universities in 1991-2 revealed that faculty, chairs, and deans found their institutions focused strongly on research, but the respondents also stated they would prefer more balance between teaching and research ( Diamond & Adam, 1998). Interestingly, those in each position viewed those in the other positions as perpetuating the bias towards research more than their own group. A follow-up study in 1996-7 surveyed 11 of the same institutions originally studied in 1991-2 ( Diamond & Adam, 1998). The follow-up study indicated a significant shift in priorities at research universities, with stronger support for balance between teaching and research in all three employee groups – it was perceived that teaching was indeed receiving more weight towards RPT than in the past. However, open-ended comments in the 1996-7 responses indicated that although a shift had occurred, policies for RPT still rewarded research more than teaching, and allocation of university resources still favored research over instruction as well.

In Tang & Chamberlain’s (1997) study of regional universities, administrators thought teaching is a crucial and rewarded activity of faculty (see also Sid & Richardson Foundation Forum, 1997), but faculty perceived that only the research component of their job requirements was actually rewarded (see also Wolfgang *et al*. 1995, for similar findings in pharmaceutical faculty). Although administrators agreed regarding the importance of research, faculty can experience a disconnect in that they feel teaching is not valued in the reward system although it is an expected activity ( Tang & Chamberlain, 1997). Wolfgang *et al*. (1995) suggested RPT policies should more accurately represent the investment faculty make in both teaching and research, with the goal of validating effort and recognizing success in both capacities.

### Perceptions of the balance between research, teaching, and service across institution type, academic position, and demographics

Gordon (2008) gave examples of faculty role conflicts, such as these comments from an assistant professor at a research university: “As a small, private university, this organization has aspirations of more research-focus. While we are supposed to focus on teaching, a colleague recently failed to receive tenure for lack of publications. This indicates to me that we are expected to produce research regardless of the school's expectations of teaching” (p. 32). Gordon observed that many faculty respondents to her survey felt tension between their roles as teacher and researcher, and developed ways to cope with this stress. Some actively gave preference to one role or the other, and others worked on their research during vacation time to meet their tenure requirements. And, the observations reported in Gordon’s study differed based on the type of institution they were collected from: faculty at research institutions reported RPT process prioritization on research-related activities, and less on teaching-related activities. Faculty at teaching institutions reported the opposite. However, faculty at hybrid institutions (those that equally value teaching and research) perceived that research was valued more than teaching, just like at research-focused institutions.

A study of information systems faculty in the late 1990s also reported tension between research, teaching, and service activities ( Whitman *et al*., 1999). Faculty from both teaching and research universities reported an overwhelming amount of service and administrative responsibility. Those at teaching institutions felt there are misconceptions that they have lower research expectations placed upon them. Rather, they feel immense pressure to publish research, often because their institution aspires to move up the ranks, which depends on its overall research productivity. Some reported feeling “victimized by this institutional pressure to achieve in research” (p. 108) alongside large teaching loads, and animosity towards their colleagues at research-oriented institutions who they believed didn't have to teach as much. On the other hand, faculty at research focused institutions expressed frustration at the assumption they don't value teaching. They reported “there has been a revival of focus on teaching … in research institutions,” and that teaching effectiveness is considered more strongly than ever in their evaluation procedures.

Whereas the aforementioned studies asked the opinions of faculty, a survey of information science department chairs conducted at about the same time asked respondents to rate the importance of research, teaching, and service in their tenure and promotion decisions ( Whitman *et al*., 1999). On a 10-point scale, research was rated 8.26, teaching 7.99, and service 5.31, showing that at least in some contexts, teaching and research are considered equally important. Several years later, Foos *et al*. (2004) reported that chairs of geoscience departments in the USA weighted teaching at 48%, research at 37%, and service at 14% in the RPT applications they evaluated. About three-quarters of department chairs rated both course evaluations and publication in national and international journals as crucial.

Despite the views expressed by these samples of department chairs, faculty continued to rate teaching as undervalued in subsequent studies. At the University of Pittsburgh School of Education, May (2005) revealed a conflict between perception of the relative weights of teaching, research, and service towards tenure and promotion versus what faculty thought should be the actual weights. Faculty estimated the actual weights used were 65.6% research, 25.6% teaching, and 8.7% service. They thought the weights should be changed to 49.3% research, 37.3% teaching, and 13.5% service to reduce emphasis on research and increase that on teaching and service. Teaching was the main target for increased focus, and faculty thought research should still contribute half to the decision making process. A few years later, Harley *et al*. (2010) still observed a corresponding focus on research and publication in RPT at the expense of teaching and service. Similarly, van Dalen & Henkens (2012) reported that faculty in high publication pressure environments, as typically experienced in the US, perceived publication in top-rank journals is the strongest factor in determining academic success.

Again, despite department chairs strongly valuing teaching towards tenure and promotion in some contexts, there remains little career advancement value in the service aspect of a faculty career. Many RPT guideline documents provide lists of research, teaching, and service requirements, but it appears “some bullet points are more equal than others” (p. 269; Macfarlane, 2007). In other words, the requirements toward research and scholarship typically outweighed those pertaining to service contributions, even if explicit weights were not given in the documentation ( Green & Baskind, 2007). For instance, University of Pittsburgh guidelines required faculty to document their service activities, but even after stating the importance of service in the evaluation, the School of Education guidelines elucidate that service on its own cannot compensate for a lack of distinguished achievement in scholarly activities such as teaching and research ( May, 2005). Thus, service is necessary but not sufficient for promotion or tenure.

Harley *et al*. (2010) came to similar conclusions from their study of research-focused universities: service and teaching “hold no weight” towards tenure and promotion in the absence of excellence in research and publication. More recent studies tend to agree. Academic pharmacy faculty in the USA raised the question of whether service was appropriately recognized in tenure review ( Pfeiffenberger *et al*., 2014). Canadian faculty similarly reported that success in tenure review depended on research, not teaching or service ( Acker & Webber, 2016). In fact, respondents reported that one’s teaching merely “need to not be horrible” (p. 239) and some even reported removing community service activities from the tenure review packages, or withdrawing from such activities altogether until after tenure. Further, some associate faculty express dissatisfaction because they are expected to devote more time to service, which takes time away from research activities that are more important for promotion ( Mamiseishvili *et al*., 2016).

Similarly, women tend to spend more time in service roles, and because service is generally undervalued in RPT evaluation, women may be disadvantaged in career advancement ( Guarino & Borden, 2017; Misra *et al*., 2011). Ethnic minorities (e.g., African Americans, Indigenous peoples) in faculty positions often face the issue of being called upon to serve on numerous institutional committees to fulfill diversity policy requirements (e.g., Henry & Kobayashi, 2017; Martinez *et al*., 2017; Ross & Edwards, 2016), leading also to more work time spent on service, taking time away from those activities valued more in career progression.

The emphasis on research and publication in the RPT process encourages faculty to focus on career advancement by conducting research of high visibility in academic circles, with less incentive to encourage dissemination of the findings to the public. Along these lines, it has been suggested that a new category be added to the RPT “trifecta” of research, teaching, and service. Harley *et al*. (2010) suggest this new category could include scholarly contributions that are generally not peer reviewed but aim to disseminate information to a wider audience, and could be considered a mid-point between service and research (see also Scheinfeldt, 2008). However, Harley *et al*. acknowledge that with few faculty including these types of contributions in their RPT packages, there is little in the way of guidelines or procedures in place for assessment. In addition, evaluating these additional materials could be time consuming and arbitrary, and the expectation for peer review may limit which contributions reviewers find meaningful.

## Quantity, quality, and prestige of publications for RPT

If it is clear that research and publications are presently the most important components of the review process, then what should academics focus on: quantity, or quality? Or is it about seeking prestige? Publications in the most prestigious venues are not necessarily those of the highest research quality; other factors such as the editors’ perceived novelty and importance of the findings also determine likelihood of acceptance for publication.

Some aspects of the evaluation of publications in RPT appear to have remained relatively consistent over the past few decades. In the 1990s and early 2000s, several studies found that those evaluating faculty for promotion or tenure preferred to focus more on the quality of their research and impact of publications as opposed to the quantity of papers ( Cronin & Overfelt, 1995; Estabrook & Warner, 2003). Department chairs liked to see “value,” “quality,” “legitimacy,” and “weight” in the publications ( Andersen & Trinkle, 2001). Publishing in peer-reviewed journals was, and remains, a key to demonstrating research quality ( Acord & Harley, 2013; Andersen & Trinkle, 2001; Cronin & Overfelt, 1995; Harley *et al*., 2010; King *et al*., 2006; Seipel, 2003) and the quality of the peer review offered by particular journals is also an important consideration ( Andersen & Trinkle, 2001).

Despite consistent value being placed on research quality and peer-reviewed publications, there is some concern that RPT research and publication requirements are gradually increasing, resulting in greater workloads and an imbalance between varied job responsibilities and the reality that faculty are expected to produce more papers and books than ever before. Estabrook & Warner (2003) provided evidence that standards for publishing in book-centric disciplines had increased based on the reports of faculty in History, English, and Anthropology departments who had received tenure. Similarly, academics in other disciplines feel pressure to publish particular numbers of articles due to RPT policy ( Walker *et al*., 2010). There can be either formal, or informal and verbally communicated, expectations regarding the number of articles required for tenure or promotion. For instance, King *et al*. (2006) found that at UC Berkeley it is typical to need three or four peer reviewed articles per year to succeed in RPT applications in biostatistics and chemical engineering. To achieve full professor in chemical engineering, one needed about twenty papers in major journals as well as widespread and international recognition in their research specialty. Similarly, Foos *et al*. (2004) reported that 27% of US geoscience departments had guidelines regarding the number of publications needed to earn tenure: the requirement was 3.7 publications on average, with a range between one and twelve.

Harley *et al*. (2010) found that across various disciplines, those assessing faculty for tenure or promotion were looking for numerous and exceptional publications that represent significant progress in their field of study, are deemed high in quality by both internal and external reviewers, and can be described as “groundbreaking,” “indicative of sustainable scholarship,” and “lauded by the larger community of scholars” (p. 7). It can be difficult to quantify exactly how many journal articles are necessary for tenure within and across different disciplines – the guidelines are not always specific, and can allow for some flexibility, especially in order to take quality into consideration.

Regarding top-tier versus second-tier institutions, Harley *et al*. (2010) found that some faculty perceived second-tier institutions to have less stringent publication requirements. The list of acceptable journals and presses was thought to be more inclusive, fewer publications were needed, and more emphasis was placed on teaching. Similarly, economics department chairs revealed that more prestigious departments required more second-tier publications to make up for the lack of publishing in a top-ten journal ( Liner & Sewell, 2009). However, some faculty in Harley *et al*.'s study thought that the requirements at top-tier research universities influenced the policies of lower-ranked institutions, with lower-ranked institutions attempting to move up the rankings by increasing their research presence.

Faculty rank can influence the career advancement process from both the applicant and the evaluation sides. On the applicant side, the evaluation process can be qualitatively different for tenure applications versus applications for promotion to full professor. Harley *et al*. (2008); Harley *et al*. (2010) found that assistant professors applying for tenure feel pressure to publish only in high-impact, high-prestige venues, whereas associate professors may publish in more varied formats, even including encyclopedias or electronic resources. It may be associate professors applying for promotion who pave the way towards inclusiveness of different media in the RPT process – this group, having already been granted tenure, has a tendency to be more open-minded towards non-traditional forms of publishing ( Harley *et al*., 2010). That being said, Liner & Sewell (2009) reported that in economics departments, those applying for promotion to full professor had to compensate more than those applying for tenure if they lacked publications in top-tier journals.

There is also evidence that shared authorship can influence the value placed on publications in RPT evaluations, which can be a cause for concern in fields of research that are increasingly collaborative in nature (e.g., Soares, 2015). Walker *et al*. (2010) found that journal article authors ranked journal impact factor, number of publications, and order of authorship as most crucial for tenure and promotion, whereas the number of authors on a paper was less of a concern. At some universities, only the first or corresponding authors received credit in the RPT process, whereas in other institutions, second and third authorship was rewarded. First or corresponding authors tended to benefit the most towards promotion, tenure, and/or financial compensation ( Mahoney, 1985; Seipel, 2003; Wren *et al*., 2007).

### Defining the quality of scholarship

There seems to be general agreement that scientific content and quality should be more important than the number of publications that are being evaluated. However, it is not always clear what constitutes a quality publication, and there is evidence that those who review RPT applications often do not directly evaluate the scientific merits of every publication listed. It is common to look at the venue of publication as a proxy for quality. This practice has been criticized, most notably in The San Francisco Declaration on Research Assessment (DORA; Cagan, 2013) and the Leiden Manifesto ( Hicks *et al*., 2015); nonetheless, evidence for this approach can be found throughout the literature.

The practice of differentiating between peer-reviewed and unreviewed publishing mediums, with peer-reviewed being the clear preference, is one method to gauge quality that is relatively uncontroversial and unchanging in recent decades. For example, in a survey sent to chairs of information science departments in the late 1990s, 43.7% reported that all journal publications count towards tenure and promotion decisions, whereas 39.2% reported that only certain categories of journal publications count, such as those that are refereed and/or editorial reviewed ( Whitman *et al*., 1999). Peer-reviewed journal articles are the main focus of evaluations in many fields, including astrophysics, biology, economics, business, psychology, women's studies, music, and some fields of political science ( Coonin & Younce, 2009; Harley *et al*., 2010; Harley *et al*., 2008).

Although determining whether a journal is peer-reviewed is fairly straightforward, RPT committees also make other distinctions that are less clear-cut. A common shortcut is to give different weights to different kinds of publications, such as those considered to be “top journals,” “prestigious,” “elite,” “impactful” or “international” ( King *et al*., 2006; Seipel, 2003; Walker *et al*., 2010). Some academic institutions even reward faculty who publish in high impact journals ( Nederhof, 2008; “The politics of science,” 2010). In geoscience departments at US universities, national and international journals scored highest at 1.22 on a scale of 1 to 5, with 1 being “Very Important” and 5 being “Not Considered” ( Foos *et al*., 2004). Book chapters and highly specific or regional journals were rated about 2, and refereed electronic journals and symposium volumes around 2.3. Ratings lower than 2.5 were given to government publications, textbooks, lab manuals, field guides, and technical reports. In another instance, both specific numbers of publications and a qualifier were used: in the field of information systems, there was an expectation of at least four articles published in “elite journals” to earn tenure ( Dennis *et al*., 2006). Taken together, these studies provide further evidence that, in terms of career success, faculty should aim to publish with as much prestige as possible, regardless of whether that represents the most appropriate medium for disseminating the work.

This evaluation strategy seems to also apply to fields that require faculty to write books or monographs as part of their tenure requirements, including music theory and history ( Harley *et al*., 2010; Harley *et al*., 2008). Like with journals, there can be standards as to what types of books and publishers are the most valuable for tenure or promotion. For example, textbooks generally contribute less towards an application than does a scholarly monograph ( Liner & Sewell, 2009). Peer-review and prestige are both of influence, with choice of publisher playing a crucial role. Books published by presses with editorial boards, or those that provide peer review of book submissions (e.g., members of the Association of American University Presses), are often weighted more heavily than those from commercial publishers ( Thatcher, 2007). UC Berkeley administrators stated that “books should be published by prestigious university presses” (p. 54), with faculty understanding this is to ensure the book is scholarly and adheres to high standards ( King *et al*., 2006). Although standards and expectations vary across institutions and fields, the studies cited above show a clear desire for rating or ranking the quality of a candidate’s contributions, something that seems to be done in large part based on the known reputation of the publishing venue (be it the journal or the publisher). 

Perhaps in an attempt to get away from the subjective nature of judging prestige, many departments have taken to using the Journal Impact Factor in assessing the value of publications towards RPT. A journal's impact factor is calculated using division: the numerator is the number of citations (in the current year) of articles published during the previous two years, and the denominator is the total number of articles published during those same two years ( Garfield, 1999). The impact factor has been widely debated and criticized, not least because of its inappropriateness for judging the quality of individual articles or researchers. Despite the well documented critiques and adverse effects (e.g., Haustein & Larivière, 2015; Hicks *et al*., 2015; Larivière & Sugimoto, 2018), the importance of the impact factor to RPT was reported across all types of faculty positions and countries surveyed by Walker *et al*. (2010). Adler *et al*.’s (2009) confidential surveys provide examples of formulas that rely on impact factors to assess publications in RPT. One example reads:

“My university has recently introduced a new classification of journals using the Science Citation Index Core journals. The journals are divided into three groups based only on the impact factor. There are 30 journals in the top list, containing no mathematics journal. The second list contains 667, which includes 21 mathematics journals. Publication in the first list causes university support of research to triple; publication in the second list, to double. Publication in the core list awards 15 points; publication in any Thomson Scientific covered journal awards 10. Promotion requires a fixed minimum number of points” (p. 10).

A second example reads:

“In our department, each faculty member is evaluated by a formula involving the number of single author equivalent papers, multiplied by the impact factor of the journals in which they appear. Promotions and hiring are based partly on this formula” (p. 10).

These examples illustrate that some institutions may see the impact factor as a convenient shortcut in assessing the research contributions of their faculty. Similarly, Malsch & Tessier (2015) report a journal rankings list used as part of their institution’s Research Incentive Policy applied in the context of determining career advancement. In this case, the authors’ field of study prohibited them from publishing in their institution’s top-ranked journals, leading to potential career consequences due to journal ranks largely based on Journal Citation Reports. Systems like this are even prompting evaluations of the usefulness of publishing in particular journals for the specific purpose of promotion and tenure (e.g., Janvrin *et al*., 2015).

Suber (2010) criticized the practice of using “journal prestige and impact as surrogates for quality” (p. 119), suggesting that it is a time saver to determine whether the journals overall are high-impact or high-prestige rather than assess the actual articles. Suber acknowledged that promotion and tenure committees can't all be experts in the candidate's field and often have to assess numerous candidates, not allowing for sufficient time to evaluate materials with the depth required to determine research quality. Even bringing in the opinions of external reviewers and experts in the candidate's discipline doesn't necessarily help the issue. External reviewers don't always have a direct connection with the candidate and may evaluate based on the apparent prestige of their publication record and how well known they are in their field ( Harley *et al*., 2010). Together, these factors suggest that evaluation of applications for promotion or tenure is a realm in which faculty may be over-stretched, which encourages use of the impact factor to gauge the quality of research publications as a way to ease workload. As a result, most faculty (e.g., 68% in medical fields) perceive journal impact factor as important to their performance review and promotion ( Walker *et al*., 2010).

If impact factors do not provide adequate information for RPT, what other indicators may be considered in the RPT process? Some institutions assess faculty’s track record of securing grant funding as part of RPT evaluation. Liner & Sewell (2009) found that in economics departments in the USA, external competitive grants generally counted towards tenure or promotion, although the size of the grant was more important in the application for full professor than it was for tenure. Securing grants is also typically important for RPT in the sciences, including biology and astrophysics ( Harley *et al*., 2010). And, Foos *et al*. (2004) found that 41% of geoscience departments in the USA require evidence of obtaining research funding in order to award tenure. However, this is not always the case. In one documented example, Duke University Medical School does not consider external funding for the promotion and tenure of clinical or basic science faculty ( Nunez-Wolff, 2007). 

## Modern approaches to evaluating research output

Numerous advances have occurred in scholarly communication over the last decades, some of which include online publication and databases, academic use of social networks, and analytic tools aimed at quantitatively assessing the reach of individual publications. Specific metrics have been developed that have the potential to reflect the influence of a candidate's publications in their field of specialty more accurately than the impact factor. Are such alternate citation measures considered in RPT evaluations?

Indeed, some institutions have begun to consider additional citation metrics, such as counts per journal article, in their decision making process ( Reinstein *et al*., 2011). Such citation searching may be required in the RPT application and it may not be an easy task for the candidate to carry out. The amount of support available from the university library varies across institutions ( Dagenais Brown, 2014), although there are freely available online resources that provide guidance in choosing and interpreting scholarly literature metrics for different situations (e.g., http://www.metrics-toolkit.org). Indeed, some have predicted there will be a movement towards using alternative metrics (altmetrics) to assess the influence of research findings for RPT ( Darling *et al*., 2013; Piwowar, 2013). Altmetrics can involve such measurements as views, discussion posts, or social media shares, of either the original research articles or other products that result from the research, such as datasets.

The idea of altmetrics is still quite new – the term itself was coined only in 2010 – and so the integration of these alternate measures of research communication with RPT processes remains in flux ( Howard, 2013). Some view altmetrics as a potentially informative addition to RPT evaluations, but there are concerns regarding the value of the data. For instance, a low-quality publication in a broadly interesting, or new and exciting field of research may generate a lot of online “buzz”, whereas a high-quality publication in a niche field may attract far less attention. Accordingly, although Gruzd *et al*. (2011) found a majority (65%) of library and information science faculty agreed that online social media use should be considered in the tenure and promotion process, most were unsure of exactly how such professional social media use should be formally evaluated. Also, 73% of faculty in this study stated that online social communication tools have significantly influenced how they use traditional information sources. This widespread, but currently informal, use of social media (including forums like Twitter, Mendeley, and blogs) has become an integral part of how some academics stay informed on progress in their fields, and can even help to accelerate the pace of scientific discovery. Despite this, only a minority (12% of faculty) in the Gruzd *et al*. (2011) study reported that their tenure and promotion procedures acknowledged so-called alternate forms of scientific communication.

Accordingly, there is little published evidence of RPT procedures directly acknowledging academic service involving outreach to the academic and public communities. In fact, Harley *et al*. (2008); Harley *et al*. (2010) found that across a number of disciplines at research-intensive institutions, pre-tenured faculty were encouraged to focus on high-impact publishing and not invest too much time on committee work, public engagement, or writing in non-traditional formats such as commentaries or blogging. Although raising scholarly visibility with blogs, working papers, or preprints may indirectly help a tenure application, Harley *et al*. (2010) reported that these items are not typically included in tenure applications, and may be considered neutral or even negative in the review process. Similarly, Goldstein & Bearman (2011) found little emphasis on community service or engagement in the RPT process at medical schools. In general, these types of activities, along with the sharing of unpublished work and using social media such as tweeting, haven't been valued by tenure and promotion committees but there is some indication this might begin to change ( Fox, 2012; Gruzd *et al*., 2011; Piwowar, 2013).

One example of scholarly social media being considered in RPT evaluation is that of the Mayo Clinic – starting in early 2016, digital portfolios were allowed in evaluations for promotion ( Cabrera *et al*., 2017). Cocchio & Awad (2014) reported that across medical, nursing, and pharmacy programs, deans have varying views regarding the value of social media in the evaluation of scholarly activity. 31% of these deans were of the opinion that high viewership of scholarly works increased academic merit, and 52% thought peer review of materials published online would also add value. It seems that the consideration of social media and altmetrics in RPT practice would be facilitated by implementing clear-cut structures for evaluation, and including the well-accepted trait of peer-review in assessing value.

Importantly, Harley *et al*. (2010) found that engagement with the public is generally valued across disciplines and by institutions. There is recognition for faculty who facilitate public education or find other ways to give back to the public as a way to acknowledge taxpayer funding. However, attempts to become a public figure aren't without their risk. Traditionally, some departments view negatively those who attempt to popularize their research niche ( “An interview with Aaron Barlow, editor of Academe, the magazine of the American Association of University Professors,” n.d.). And, some academics view high levels of public engagement as only appropriate for those who have already been granted tenure and are well known to academics in their field; faculty may garner criticism if their public persona is not balanced with significant research contributions ( Harley *et al*., 2010). However, Aaron Barlow argues that any academic who has succeeded in having their work taken seriously by the public is likely to also be taken seriously in RPT ( “An Interview with Aaron Barlow,” n.d.).

It has also been suggested that universities should shift to formally recognizing the translational value of academic research in the RPT process ( Sanberg *et al*., 2014). In general, about half of faculty agree that the societal impact of one’s scholarly work should be a key RPT consideration ( Wolff *et al*., 2016). Specifically, patents, licensing, and commercialization could be credited in order to encourage faculty to engage in use-oriented research that has the potential to positively affect society. Further, about 35% of faculty believe data should be credited equally to academic publications in RPT evaluations, and 37% believe software/code should be equally credited ( Wolff *et al*., 2016). Sanberg *et al*. (2014) report that an apparent minority of US institutions have integrated these ideas into RPT policy, and changes in policy on this theme have likely been slow because they have been initiated primarily at the level of individual departments (bottom-up) rather than that of the institution (top-down).

## Beyond RPT guidelines

To reform the RPT process, it might be logical to begin by examining how the process has been instituted in formal guidelines. However, it seems that RPT guidelines can be unclear ( Smesny *et al*., 2007), or purposefully vague, to allow for flexibility in each applicant's situation. Although promotion and tenure committees usually do attempt to use objective measures, in reality, the procedures, criteria, and weights used can vary between applicants and between departments ( Claxton, 2005; Walker *et al*., 2010). Macfarlane (2007) observed that institutions typically don't specify weights to convey which of their tenure and promotion criteria are the most important. May (2005) found that all promotion and tenure documents from several research-focused universities addressed research, teaching, and service, but the language of the policy tended to be very broad as to allow for interpretation. All documents that May reviewed had specific requirements with regard to publication of research findings, but the expectations for teaching were less clear and more variable, and the definitions of service requirements were the most vague.

Faculty rank and institution type may also affect the way one views the RPT process itself. Diamantes (2004) reported that tenured faculty perceived that the requirements were well communicated, but untenured faculty expressed a degree of uncertainty regarding the expectations. Estabrook & Warner’s (2003) study on Anthropology, English, and History departments, however, found no relationship between faculty age or tenure status and opinion on whether a book should be required for tenure. And, Gordon (2008) found that faculty at research and hybrid (research/teaching) universities report less confusion about publishing requirements than faculty at teaching universities. She provided different examples of faculty from research universities who had specific guidelines for publishing (e.g., six publications in six years) versus guidelines that were difficult to interpret, as one respondent wrote: “It is 45% of my responsibility allocation, but I’m not sure that tells the whole story. I think its more that I need to have quality or quantity of pubs. I’m not sure how they can translate that into a percentage” (p. 64). King *et al*. (2006) described the RPT process in chemical engineering at UC Berkeley as having vague and ambiguous written guidelines – even requirements for publication were not clearly stated.

Despite ambiguous guidelines, faculty in King *et al*.’s (2006) study reported a clear understanding of how to succeed in career advancement, indicating the value of informal communication within the department in supporting its members. In survey responses, faculty expressed the opinion that vague requirements are understandable because the RPT process is “unquantifiable,” and that “if I'm doing my job right, tenure should come along with it” (p. 39; King *et al*., 2006). Acker & Webber (2016) similarly reported that in Ontario, Canada, many candidates found the rules for tenure criteria lacking in clarity. And, Prottas *et al*. (2017) found that faculty in the northeastern USA experienced a lack of clarity, and perceived unfairness, in their tenure criteria and in their institutions’ decision making processes. In the UC Berkeley Anthropology Department, it was acknowledged that the process for career advancement can be unclear to junior faculty, therefore it is the responsibility of the department chair to explain tenure expectations to new hires ( King *et al*., 2006).

Harley *et al*. (2010) also received reports of considerable flexibility in tenure and promotion judgement at research universities. Excellent quality in research and publication was most important and could override unwritten rules about the numbers of journal articles, books, or citations required. Special forms of scholarly evidence, such as the products of interdisciplinary research, creative pursuits, and many practices more common in the arts, can require special attention by reviewers. Harley *et al*. noted that RPT policy had built-in mechanisms to credit these types of activities as appropriate, and that each RPT application receives a great deal of attention in its adjudication. May (2005) concluded that the paucity of particular weights or values for any particular aspect of tenure or promotion applications leads to decisions being made by individuals and committees using their own “weighted judgement for each given criteria,” or by viewing all evidence together to make a prediction about the applicant's potential for making ongoing and substantial scholarly contributions.

Estabrook & Warner (2003) also found evidence of tenure and promotion committees deviating from policy in making career advancement decisions in the somewhat variable disciplines of Anthropology, English, and History. Here, it is generally expected that faculty members will have published a scholarly book or monograph prior to making a tenure application. However, most official promotion and tenure guidelines indicate that either a book or a considerable number of substantial and peer reviewed publications may be accepted. When Estabrook and Warner interviewed 17 department chairs in these disciplines, the chairs consistently acknowledged the option given in the RPT guidelines, but stated that most faculty (with the exception of those in a few specific subfields) needed to publish a book to receive tenure.

One way to describe the relationship between RPT policy and the way the RPT process is actually carried out is to acknowledge the difference between institutional policy and departmental expectations. Institutional policy tends to be broad with many potential criteria for faculty to meet in order to earn career advancement. Departments may pick and choose from the institutional framework which criteria are their particular deal-breakers, and which items can be overlooked in favor of other candidate qualities and contributions. This, of course, can also lead to differences in the RPT process not only between institutions, but between departments within the same institution, as reported by Andersen & Trinkle (2001).

Just as departments within an institution can vary in their RPT practices, so can departments of the same discipline across different institutions. For instance, Liner & Sewell (2009) surveyed 125 economics department chairs regarding their consideration of applications for both tenure and advancement to full professor, and found variability between them in the degree of reduction of credit for paper co-authorship. Reports from faculty in the field of English-language literature echo this theme, with one faculty member in King *et al*.’s (2006) study stating that the norms for advancement in the field “vary wildly” (p. 23). Although the general opinion was that across institutions the differences were substantive, faculty were clear on what was required within their own institution. Overall, it seems that policies provide a framework, but that RPT decisions are made on a case-by-case basis with considerable allowances made for differences from the norm.

## Conclusions: The future of RPT

Expectations and practices for review, promotion and tenure have shifted significantly over the last few decades. Although there are differences across institutions, disciplines, and faculty ranks, it is clear that faculty in many contexts are feeling increasing pressure to focus on research at the expense of teaching and service ( Otten *et al*., 2015; van Dalen & Henkens, 2012). In recent years there has been an effort to help the pendulum swing back the other way by allowing for consideration of more varied measures of performance (e.g., altmetrics or non-traditional publishing mediums), but these efforts have not been entirely successful in offsetting oversimplified approaches such as points schemes based on journal impact factors. As a result, those faculty who wish to value activities beyond traditional research publications in so-called high-prestige venues may face barriers to career advancement.

Although there are frustrations with RPT practices, this doesn’t mean the RPT process is fixed as it is today. The noticeable shift towards greater emphasis on research and particular types of publications, along with the documented efforts to counteract those trends, are signs that RPT practices do not go uncontested. Part of this challenge to the current status-quo was the San Francisco Declaration on Research Assessment (DORA), drafted at the Annual Meeting of The American Society for Cell Biology in December 2012 ( Cagan, 2013) and since signed by over 450 organizations and almost 12,000 individuals ( DORA, n.d.). The declaration makes several recommendations that are directly aimed at pushing back on some of the trends in researcher assessment highlighted in this review. In particular, they recommend, among other things, that researchers, and those involved in assessing research: 1) “Do not use journal-based metrics, such as Journal Impact Factors, as a surrogate measure of the quality of individual research articles, to assess an individual scientist’s contributions, or in hiring, promotion or funding decisions;” 2) “Be explicit about the criteria used to reach hiring, tenure and promotion decisions, clearly highlighting, especially for early-stage investigators, that the scientific content of a paper is much more important than publication metrics or the identity of the journal in which it was published;” and 3) “When involved in committees making decisions about funding, hiring, tenure, or promotion, make assessments based on scientific content rather than on publication metrics.” The global effect of these recommendations on changing the current RPT practices, however, remains largely unknown.

DORA has inspired much updating of policy and shifting of opinions away from the use of journal impact factors, but there is still a great need for action to elicit change in the actual procedures used in RPT evaluations. In working with DORA, Curry (2018) has observed numerous instances of RPT procedures that are maintaining the dominance of the impact factor in determining the value of research. The next step will focus on moving beyond declarations and focusing on finding ways for institutions and funding agencies to change their evaluation practices in the spirit of the declaration ( Curry, 2018).

Although DORA is promoting change in procedures for evaluation of academic research contributions, the issue of imbalance within the academic “trifecta” of research, teaching, and service remains. Faculty seem accepting of the idea that research may count more towards RPT than the other two elements, but failure to reward teaching and service devalues faculty work in these areas. It may be time to evaluate whether our institutions of higher education and mechanisms of scholarly communication can reflect Boyer's (1996) *scholarship of engagement*, in which scientific discovery (research) is a crucial function of the university, but so are functions deriving from teaching and service, such as the sharing of information across disciplines, the sharing of knowledge with students and the public, and the application of information to real world problems.

Because RPT criteria strongly influence where faculty will place their focus, RPT reform may be one of the most successful ways to effect change in the academic system. We believe there are two natural best next steps to devising an updated system for evaluating scientific merit: 1) to deepen our understanding of faculty and administrative perceptions of the current reward system and desires moving forward (see also Desrochers *et al*., 2018); and 2) to assess the relationship between the content of current RPT documents and their actual operationalization into existing practices. Together with the foundation of information presented in this review, progress in these directions will provide insight into how RPT should be reformed, and whether there may be additional targets for change within the academic system.

## Data availability

No data is associated with this article.
