# Supplementary material for: The evaluation of scholarship in academic promotion and tenure processes: Past, present, and future
Source: F1000Res. 2018 Oct 5;7:1605. [Version 1] doi: 10.12688/f1000research.16493.1 (PMC6325612; doi:10.12688/f1000research.16493.1)
Supplement: Peer review history

# Peer review: Referee response for version 1

**DOI:** 10.5256/f1000research.18027.r42353

**Published:** 2019-01-04

**Authors:** Aaron Barlow

**Affiliations:** 1 New York City College of Technology (CUNY), New York City, NY, USA

This article is sound as it is. In fact, it is more than sound; it is an excellent presentation of an important contemporary issue in higher education. The questions raised below are not of what needs to be addressed in the article but that need to be addressed following it.

 The questions surrounding Reappointment Promotion Tenure (RPT) in contemporary colleges and universities need to be addressed with care, compassion and comprehensively. This article is a good start toward focusing ongoing discussion in a way that can be useful across the range of Canadian and American institutions of higher education. There are a number of areas I would like to see more exploration of, but I think they are going to require further research and consideration; there is certainly not room for them here though the article does point to them.

 One of the areas is the relative importance of teaching, scholarship and service in different institutional situations. Where there is great reliance on adjuncts for teaching, for example, service takes on an importance it may not have when there is adequate tenured and tenure-track faculty to cover departmental needs. Where students are reaching college with inadequate preparation, also, an emphasis on teaching may be more important than in a situation of selective admissions. These points are recognized in the article, certainly, but a great deal more consideration is warranted.

 Also touched on but needing a great deal more exploration (though, again, in future articles) is what amounts to the lumping of various scholarship needs and standards in various fields. This is mentioned (the emphasis on books in certain fields, for example) but the history behind the movement toward a focus on peer review and other commonalities between fields is worthy of careful research. My suspicion is that it goes back at least to reaction to C. P. Snow’s *The Two Cultures *in the late 1950s when the humanities and social sciences began to model themselves after scientific disciplines. What should “scholarship” mean in different fields?

 The article does present the idea, originating elsewhere, that a fourth category be added to scholarship, teaching and service, one that gives credit for scholarly and popularization work that is not peer reviewed but that benefits the community. This is another area that could lead to fruitful discussion, though the upshot might not be a fourth area but instead a broadening of what is considered scholarship in institutional settings.

 It could also be fruitful, in terms of re-evaluating RPT, to see new and more specific historical studies presenting snapshots of RPT as it was practiced 25, 50, 75 and 100 years ago. American universities built their reputations over the last century; it would be interesting to see how tenure and promotion processes worked for past generations.

 Finally, I would like to see the essence of this article distilled into another piece that succinctly outlines the questions and possibilities raised in a way that can be used in departments with little interest in the specifics of the excellent research presented here but who need to be reassessing RPT procedures in light of changed teaching, scholarship and service environments.

 Obviously, the subject of this article is worthy of a book. I hope, therefore, that the authors continue down the paths they explore here. Without national consideration of contemporary concerns relating to RPT in both Canada and the United States, problems such as predatory publishing, among others, will not only continue but will expand.

I have read this submission. I believe that I have an appropriate level of expertise to confirm that it is of an acceptable scientific standard.

# Peer review: Referee response for version 1

**DOI:** 10.5256/f1000research.18027.r40287

**Published:** 2018-11-12

**Authors:** Jonathan P. Tennant

**Affiliations:** 1 Open Science MOOC, Berlin, Germany; 2 IGDORE, Berlin, Germany

The authors present a non-systematic review of review, promotion and tenure (RPT) guidelines, including the diversity of practices, historical context, and recent and ongoing developments happening in the world of scholarly communication. This effort is valuable due to the importance of these processes in the wider scholarly communication context, and their influence on cultural and social shifts within the world of scholarly research. The article is well-written, and I believe very timely given the importance of this debate within scholarly communications at the present.

 My relevant expertise in reviewing this manuscript comes from being a researcher interested in developments in scholarly communication, of which issues to do with careers and incentives come up frequently. As such, I have a vested interest in seeing that research like this is published and widely communicated to stimulate further discussion on critical topics such as this.

**Basic reporting:**

- There do not appear to be any figures or data accompanying this manuscript. While not inherently problematic, I wonder if there are any potential images to go in this paper, simply to help break up the text for readers?
- The article is well-written, and should be of interest to both generalist and specialist audiences.

**General comments:**

- I noted several instances in the Abstract where the language was not as clear as it could be. A thorough copy editing is required before the manuscript is accepted for indexing.
- Concerning structure, the Introduction section finishes with the goals of the research, before launching into a ‘Previous Research’ section as a literature review. This section should be integrated into the Introduction section, with the whole section finishing with the aims/goals immediately before the Methods.

**Abstract:**

- The abstract is concise and conveys the context and main findings of the research. No key bits seem to be missing. I wonder, if there is space, if a final sentence could be provided just to reiterate the importance and potential impact of this work (similar to the opening sentence).

**Introduction**

 P1:

- Problems with peer review/reproducibility are more about research than ‘academia’, if you want to be niggly.
- Has anyone studied RPT guidelines and their impact on academia before in an empirical manner? I wonder if the paper of the authors recently published (Alperin *et al.*, 2018 ^1^) should be cited here, to make the link between these two clearer?

P2:

- For Buttliere’s quote, is it worth emphasizing here that thus the present incentives often encourage research to be conducted in ways that are not in the best interests of research, and the wider impact of research on society?
- Just for the sake of context, could you perhaps explain why the USA/Canada were singled out for this study? Is it just for the sake of simplicity and scope, or because there are things that are inherently different about these countries?

**Identifying RPT issues and areas for reform**

 P1:

- Re. the Diamond and Adam reference, is this experience based across all types of universities and disciplines, or is it more specific? What are ‘service activities’ in this context too? Actually, the same for the second point too. Are these universal concerns, or are there large areas where we actually don’t have any understanding of how faculty perceive these issues? I suspect that there are probably large gaps in our knowledge here.
- Here, it might help to briefly discuss who actually drafts these RPT guidelines?

P2:

- I wonder if it’s worth noting that this link even has its own mantra, ‘publish or perish’, just to gain some familiarity with the issue.
- Perhaps it is also worth noting here the growth of Open Access publishing in recent years (Piwowar *et al.*, 2018 ^2^), and the relatively poor understanding that we have on its potential impact on hiring and decision-making processes? Perhaps the fact that OA has come from a combination of bottom-up and top-down approaches seems to have created a lot of uncertainty in this space, and an apparent tension between developments in publishing and career advancement, is worth noting here too. As this does also highlight the importance for this study.

**Research, teaching, and service in the review process**

 P1:

- Just a note here, is tenure something one seeks to attain in all countries? And on that note, I haven’t checked all the references within, but are they focused on the USA/Canada, or are some more broad or based on different geographic systems? I just wonder if it should be made clearer, as it might be a little confusing in a paper about the USA/Canada if some of the evidence being cited is based on a different geographic region.
- Another thought. This is a non-systematic review, correct? Could the authors perhaps comment on how they selected the articles for this review? I’m not by any means an expert of the literature, and cannot tell from an objective point of view if the articles discussed within are representative or not of the total literature on this topic.

P2:

- I feel that the detailed criticism of ‘excellence’ by Moore *et al.* (2017 ^3^) should be mentioned here for important additional context.
- ‘Surveyed over’ – typo, space missing. Very important, I know.

**Perceptions of the shift towards prioritizing research in career advancement**

 P1:

- I wonder here if it is worth noting the article by McKiernan *et al.* (2016 ^4^) which makes a strong case for ‘open’ research practices being beneficial to the career of an individual researcher? I think this fits in because it is sort of a different ‘type’ of research style that is becoming influential, perhaps.

**Perceptions of the balance between research, teaching, and service across institution type, academic position, and demographics**

 P1:

- I really like this discussion, and all of it so far. It’s all relevant, important, and ties into the theme of the paper without any waffle. I wonder though if it is worth discussing more the potential consequences of these perceptions though. For example, the seeking of high impact journals, salami slicing of publications, questionable research practices, the impact on the very social culture of academia, only researching topics perceived to be of high interest rather than academic importance. All of these things seem to be related to the increasingly performance-driven research system in some way.

**Quantity, quality, and prestige of publications for RPT**

 P1:

- I think perhaps it is worth clarifying here what prestige means in this context. Are quantity, quality, and prestige all independent too? Potential Venn diagram alert here.
- I wonder if here it would be a good idea to cite some of the work by Björn Brembs (Brembs, 2018 ^5^ and Brembs *et al.* (2013 ^6^) on issues to do with journal prestige? It seems potentially relevant to readers.

P2:

- Could you expand on what is meant by ‘impact’ here? Or any of the other descriptive terms? Or are they just vague? Is there any research out there that shows that peer review affects research quality (positively or negatively) that could be used to enhance this discussion here?

P3:

- For Foos *et al. *(2004), is that per year or in total before attaining tenure?
- I think at some point in this section, it needs to be noted that the focus on peer reviewed scholarly research articles as primary outputs for assessment is a ridiculously discriminatory process; for example, against data collectors/managers, software engineers, lab technicians (etc.) that are critical for the process, but can often be excluded from final publication author lists.

P7:

- What might some of the consequences be of this prioritization of authorship? How might it affect the ways in which authorship orders are determined?

**Defining the quality of scholarship**

 P1:

- First sentence, citation needed.

P2:

- Do you think this binary state of peer reviewed versus non-peer reviewed in demarcating quality is appropriate, and evidence-based? You don’t have to cite me on this, but I have particular issues with this black and white approach to quality differentiation, and feel there are better ways it can be done (Tennant, 2018 ^7^).
- What are the ‘certain categories’ here? I see it is explained in the next paragraph, but could be linked better perhaps.

P3:

- By ‘reward faculty’ here, do you mean beyond giving them a career?
- How do you define ‘appropriate’ here? For the wider research community, for the wider public, to align with the mission of the research institute?
- What impact does the hunt for prestige have on new entrants to the scholarly publishing ‘market’?
- Could you possibly discuss where this demand for prestigious publications comes from? Surely this was not always the case? Does it have any impact on academic culture, research practices, and public mission of universities?

P5:

- Is the IF calculation that simple? Can it be replicated? Are the data open? Who controls the data, and are they biased in any way? Are some negotiated? For such an important statistic, I think these things might need commenting on.
- Need to just clarify that the inappropriateness is due to the ‘level’ of the proxy and the lack of correlation between this metric and the level that it is often used for in assessments. And also, perhaps that this was never its intended use. I feel that there is a certain preprint that could be cited here too.
- I wonder if it is worth further commenting on the fact that the use of the IF in such a manner is a profoundly non-scientific practice, has little basis in reason, and yet seems to be one of the defining features in governing modern academic culture.

P6:

- ‘Shortcut’ compared to what?

P7:

- This is a really important piece of discussion, and one which comes up over and over again in defense of using the impact factor. Could the authors possibly comment on some potential solutions to this pain point, from their point of view? (I see the next paragraph touches on this a bit).

**Modern approaches to evaluating research output**

 P1:

- Could a couple of examples of each be mentioned here?
- Some discussion of potentially novel ways to provide incentives and reputation are given here again too, largely based on utilization of academic social networks (Tennant, 2018 ^7^).

P3:

- Maybe worth discussing some of the ideas that revolve around altmetrics and social impact here. And also the distinction between an altmetric number (e.g., like via Altmetric), and the utility of the context that comes with this?

P4:

- Was the lack of focus on other things than high impact publications here explicit?
- Are there any more recent studies on this issue than Harley *et al.*? In eight years there seems to have been a lot of changes on this topic, although perhaps not well studied. I think both DORA and ASAPbio at least have anecdotal data that might be useful context here.

P7:

- Could you describe what is meant by ‘societal impact’ here? And perhaps how traditional publishing, new forms of communication, and altmetrics fit into this concept.

**Beyond RPT guidelines**

 P6:

- Has anyone besides Estabrook and Warner (2003) ever conducted a study into the relationship between RPT guidelines and the actual practices of those involved in the process? Is this a major gap in our understanding here?

**Conclusions**

 P2:

- I feel some credit should be given to the Leiden Manifesto here too. Are there any other initiatives that warrant mentioning too?

P3:

- Is there any evidence as to whether DORA has had a true impact or not? I see the Curry (2018) article is cited here, but perhaps some explicit examples can be given. I know it is beyond the USA/Canada, but perhaps developments with ‘Plan S’ can also be cited here, with their recommendations to follow DORA or an equivalent (also includes the Gates Foundation now as a USA-based funder).

P4:

- Do you feel the imbalance of this trifecta is perhaps one of the causes behind a general system of inertia towards fairer, or more rigorous, research(er) evaluation processes?

P5:

- What might some of the wider impacts of these two steps be within the present and future system of research?

Congratulations to the authors on a great piece of work, and I look forward to seeing their research published in a revised form at some point. Please note that virtually all of the comments here are simply questions or comments to improve the argumentation style and narrative of the paper, which in my view is otherwise sound and a valuable contribution to the scholarly record.

 Sincerely,

 Jonathan Tennant

I have read this submission. I believe that I have an appropriate level of expertise to confirm that it is of an acceptable scientific standard.

## References

[1] AlperinJPFischmanGEMcKiernanECNievesCMNilesMTSchimanskiL: How significant are the public dimensions of faculty work in review, promotion and tenure documents?. *Humanities Commons [preprint]*.2018; http://dx.doi.org/10.17613/M6W950N35 10.17613/M6W950N35 Reference source PMC639106330747708

[2] PiwowarHPriemJLarivièreVAlperinJPMatthiasLNorlanderBFarleyAWestJHausteinS: The state of OA: a large-scale analysis of the prevalence and impact of Open Access articles. *PeerJ*.2018;6: 10.7717/peerj.4375 e4375 10.7717/peerj.4375 29456894PMC5815332

[3] MooreSNeylonCPaul EveMPaul O’DonnellDPattinsonD: “Excellence R Us”: university research and the fetishisation of excellence. *Palgrave Communications*.2017;3: 10.1057/palcomms.2016.105 10.1057/palcomms.2016.105

[4] McKiernanEBournePBrownCBuckSKenallALinJMcDougallDNosekBRamKSoderbergCSpiesJThaneyKUpdegroveAWooKYarkoniT: How open science helps researchers succeed. *eLife*.2016;5: 10.7554/eLife.16800 10.7554/eLife.16800 PMC497336627387362

[5] BrembsB: Prestigious Science Journals Struggle to Reach Even Average Reliability. *Front Hum Neurosci*.2018;12: 10.3389/fnhum.2018.00037 37 10.3389/fnhum.2018.00037 29515380PMC5826185

[6] BrembsBButtonKMunafòM: Deep impact: unintended consequences of journal rank. *Front Hum Neurosci*.2013;7: 10.3389/fnhum.2013.00291 291 10.3389/fnhum.2013.00291 23805088PMC3690355

[7] TennantJP: The state of the art in peer review. *FEMS Microbiol Lett*.2018;365(19) : 10.1093/femsle/fny204 10.1093/femsle/fny204 30137294PMC6140953
